# The Law Relating to Infectious Diseases.—IV

**Published:** 1902-08-30

**Authors:** J. E. R. Stephens

**Affiliations:** Barrister-At-Law, Author of "Digest of Public Health Cases," etc. etc.


### THE INSTITUTIONAL WORKSHOP.

### THE LAW RELATING TO INFECTIOUS DISEASES.—IV.

By J. E. R. STEPHENS, Barrister-at-law, Author of "Digest of Public Health Cases," etc. etc.

### DAIRIES.

BY sect. 4 of the Infectious Diseases (Prevention) Act, 1890, where that Act has been adopted, it is enacted that in case the medical officer of health is in possession of evidence that any person in the district is suffering from infectious disease attributable to milk supplied within the district from any dairy situate within or without the district, or that the consumption of milk from such dairy is likely to cause infectious disease to any person residing in the district, such medical officer shall, if authorised in that behalf by an order of a justice having jurisdiction in the place where such dairy is situate, have power to inspect such dairy, and, if accompanied by a veterinary inspector or some other properly qualified veterinary surgeon, to inspect the animals therein, and if on such inspection the medical officer of health shall be of opinion that infectious disease is caused from consumption of the milk supplied therefrom, he shall report thereon to the local authority, and his report shall be accompanied by any report furnished to him by the said veterinary inspector or veterinary surgeon, and the local authority may therefore give notice to the dairyman to appear before them within such time—not less than 24 hours—as may be specified in the notice, to show cause why an order should not be made requiring him not to supply any milk therefrom within the district until such order has been withdrawn by the local authority; and if, in the opinion of the local authority, he fails to show such cause, then the local authority may make such order as aforesaid, and the local authority shall forthwith give notice of the facts to the sanitary authority and county council (if any) of the district or county in which such dairy is situate, and also to the Local Government Board. An order made by a local authority in pursuance of this section shall be forthwith withdrawn on the local authority, or the medical officer of health on its behalf, being satisfied that the milk-supply has been changed, or that the cause of the infection has been removed. Any person refusing to permit the medical officer of health, on the production of such order as aforesaid, to inspect any dairy, or, if so accompanied as aforesaid, to inspect the animals kept there, or after any such order not to supply milk as aforesaid has been given, supplying any milk within the district in contravention of such order, or selling it for consumption therein, shall be deemed guilty of an offence against this Act. Proceedings in respect of such offence shall be taken before the justices of the peace having jurisdiction in the place where the said dairy is situate. No dairyman shall be liable to an action for breach of contract if the breach be due to an order from the local authority under this Act.

Before the year 1886 sanitary authorities, as such, had no powers or duties in relation to this matter, the administration of the Contagious Diseases (Animals) Act, 1878, and of the orders made thereunder by the Privy Council being vested in boroughs in the town councils acting under the Municipal Corporations Act, 1882, and in other areas outside the Metropolis, in the county authorities. Section 9 of the Contagious Diseases (Animals) Act, 1886, however, provided that the powers vested in the Privy Council of making general or special orders under section 34 of the Contagious Diseases (Animals) Act, 1878, should thenceforth be exercisable by the Local Government Board, who might from that time alter or revoke any such order; and that, for the purposes of these two sections and any order in force thereunder, the expression "local authority" should (unless the context otherwise required), outside the Metropolis, mean the urban or rural sanitary authority as the case may be.

Sect. 34 of the Act of 1878 gave power to the Privy Council to make orders for the following purposes:—(1) For the registration with the local authority of all persons carrying on the trade of cowkeepers, dairymen, or purveyors of milk. (2) For the inspection of cattle in dairies and for prescribing and regulating the lighting, ventilation, cleansing, drainage, and water-supply of dairies and cowsheds in the occupation of persons following the trade of cowkeepers or dairymen. (3) For securing the cleanliness of milk-stores, milk-shops, and of milk-vessels used for containing milk for sale by such persons. (4) For prescribing precautions to be taken for protecting milk against infection or contamination. (5) For authorising a local authority to make regulations for the above purposes or any of them, subject to such conditions, if any, as the Privy Council might prescribe.

In pursuance of this section the Privy Council on June 15th, 1885, made an order known as "The Dairies, Cowsheds and Milkshops Order of 1885," which, modified by the Act of 1886, and an order of the Local Government Board of November 1st, 1886, is still in force.

Article 6 of the Order provides that it shall not be lawful for any person to carry on in the district of any local authority the trade of cowkeeper, dairyman, or purveyor of milk, unless he is registered as such in accordance with this article. Every local authority shall keep a register of persons from time to time carrying on in their district the trade of cowkeepers, dairymen, or purveyors of milk, and shall from time to time revise and correct the register. The local authorities shall register every such person, but the fact of such registration shall not be deemed to authorise such person to occupy as a dairy or cowshed any particular building, or in any way precludes any proceedings being taken against such person for non-compliance with or infringement of any of the provisions of this Order or any regulations made thereunder. The local authority shall from time to time give public notice by advertisement in a newspaper circulating in their district, and if they think fit by placards, handbills, or otherwise, of registration being required, and of the mode of registration.

Article 7 deals with the construction and water-supply of new dairies and cowsheds. It provides (1) That it shall not be lawful for any person following the trade of cowkeeper or dairyman to begin to occupy as a dairy or cowshed any building not so occupied at the commencement of the Order (*i.e.*, June 30th, 1885), unless and until he first makes provision, to the reasonable satisfaction of the local authority, for the lighting and the ventilation, including air-space, and the cleansing, drainage, and water-supply of the same, while occupied as a dairy or cowshed; and (2) That it shall not be lawful for any such person to begin so to occupy any such building without first giving one month's notice in writing to the local authority of his intention so to do.

Article 8 contains provisions with respect to the sanitary state of all dairies and cowsheds. It provides that it shall not be lawful for any person following the trade of cowkeeper or dairyman to occupy as a dairy or cowshed any building, whether so occupied at the commencement of this Order or not; if and as long as the lighting and the ventilation, including air-space, and the cleansing, drainage, and water-supply thereof are not such as are necessary or proper (*a*) For the health and good condition of the cattle therein; and (*b*) For the cleanliness of milk-vessels used therein for containing milk for sale; and (*c*) For the protection of the milk therein against infection or contamination.

Articles 9, 10, 11, and 12 deal with the protection of milk from contamination or infection. They provide as follows:—(9) It shall not be lawful for any person following the trade of cowkeeper or dairyman or purveyor of milk, or being the occupier of a milk-store or milk-shop—(*a*) to allow any person suffering from a dangerous infectious disorder, or having recently been in contact with a person so suffering, to milk cows or to handle vessels used for containing milk for sale, or in any way to take part or assist in the conduct of the trade or business of the cowkeeper or dairyman, purveyor of milk, or occupier of a milk-store or milk-shop, so far as regards the production, distribution, or storage of milk; or (*b*) if himself so suffering or having recently been in contact as aforesaid, to milk cows, or handle vessels used for containing milk for sale, or in any way to take part in the conduct of his trade or business, as far as regards the production, distribution, or storage of milk, until in each case all danger therefrom of the communication of infection to the milk or of its contamination has ceased. (10) It shall not be lawful for any person following the trade of cowkeeper or dairyman, or purveyor of milk, or being the occupier of a milk-store or milk-shop, after the receipt of notice of not less than one month from the local authority calling attention to the provisions of this article, to permit any water-closet, earth-closet, privy, cesspool, or urinal to be withir, communicate directly with, or ventilate into, any dairy or any room used as a milk-store or milk-shop. (11) It shall not be lawful for any person following the trade of cowkeeper or dairyman or purveyor of milk, or being the occupier of a milk-store or milk-shop, to use a milk-store or milk-shop in his occupation, or permit the same to be used as a sleeping apartment or for any purpose, incompatible with the proper preservation of the cleanliness of the milk-store or milk-shop, and of the milk vessels and milk therein, or in any manner likely to cause contamination of the milk therein. (12) It shall not be lawful for any person following the trade of cowkeeper or dairyman or purveyor of milk, to keep any swine in any cowshed or other building used by him for keeping cows, or in any milk-store or other place used by him for keeping milk for sale.

Article 13 enables a local authority from time to time to make regulations for the following purposes or any of them:—(*a*) For the inspection of cattle in dairies; (*b*) for prescribing and regulating the lighting, ventilation, cleansing, drainage, and water-supply of dairies and cowsheds in the occupation of persons following the trade of cowkeepers or dairymen; (*c*) for securing the cleanliness of milk-stores, milk-shops, and of milk-vessels used for containing milk for sale by such persons; (*d*) for prescribing precautions to be taken by purveyors of milk and persons selling milk by retail against infection or contamination.

By Article 14 the following provisions shall apply to regulations made by a local authority under this Order:—(1) Every regulation shall be published by advertisement in a newspaper circulating in the district of the local authority; (2) the local authority shall send to the Local Government Board a copy of every regulation made by them not less than one month before the date named in such regulation for the same to come into force; (3) if at any time the Local Government Board are satisfied, on inquiry with respect to any regulation, that the same is of too restrictive a character, or otherwise objectionable, and direct the revocation thereof, the same shall not come into operation, or shall thereupon cease to operate, as the case may be.

Article 15 provides with respect to the existence of disease among cattle, that, if at any time disease exists among cattle in a dairy or cowshed or other building or place, the milk of a diseased cow therein—(*a*) shall not be mixed with other milk; and (*b*) shall not be sold or used for human food; and (*c*) shall not be sold or used for food of swine or other animals, unless and until it has been boiled.

The penalties imposed for offences against the Order of 1885 are £5 for every such offence, and in the case of continuing offences an additional penalty of £2 for every day after written notice of the offence from the local authority. Power is, however, given to the justices or court before whom any complaint is made, or proceedings taken, to reduce the amounts of these penalties.

### Disinfection of Public Conveyances.

Section 127 of the Public Health Act, 1875, provides that every owner or driver of a public conveyance shall immediately provide for the disinfection of such conveyance after it has to his knowledge conveyed any person suffering from a dangerous infectious disorder; and if he fails to do so he shall be liable to a penalty not exceeding £5; but no such owner or driver shall be required to convey any person so suffering until he has been paid a sum sufficient to cover any loss or expense incurred by him in carrying into effect the provisions of this section.

Section 126 imposes a penalty on persons who enter a public conveyance while suffering from a dangerous infectious disorder.

